# Nonreciprocity in CHIKV and MAYV Vaccine-Elicited Protection

**DOI:** 10.3390/vaccines12090970

**Published:** 2024-08-27

**Authors:** Whitney C. Weber, Takeshi F. Andoh, Craig N. Kreklywich, Zachary J. Streblow, Michael Denton, Magdalene M. Streblow, John M. Powers, Gauthami Sulgey, Samuel Medica, Igor Dmitriev, David T. Curiel, Nicole N. Haese, Daniel N. Streblow

**Affiliations:** 1 Vaccine and Gene Therapy Institute, Oregon Health and Science University, Beaverton, OR 97006, USA; weberw@ohsu.edu (W.C.W.); andot@ohsu.edu (T.F.A.); kreklywi@ohsu.edu (C.N.K.); strebloz@ohsu.edu (Z.J.S.); dentonm@ohsu.edu (M.D.); jpowers@unc.edu (J.M.P.); sulgey@ohsu.edu (G.S.); medica@ohsu.edu (S.M.); nnhaese@gmail.com (N.N.H.); 2 Department of Molecular Microbiology and Immunology, Oregon Health and Science University, Portland, OR 97239, USA; 3 Cancer Biology Division, Department of Radiation Oncology, Washington University, St. Louis, MO 63110, USA; idmitriev@wustl.edu (I.D.); dcuriel@wustl.edu (D.T.C.); 4 Division of Pathobiology and Immunology, Oregon National Primate Research Center, Beaverton, OR 97006, USA

**Keywords:** Chikungunya virus (CHIKV), Mayaro virus (MAYV), alphaviruses, vaccine, cross-neutralization, antibody

## Abstract

Chikungunya virus (CHIKV) is a pathogenic arthritogenic alphavirus responsible for large-scale human epidemics for which a vaccine was recently approved for use. Mayaro virus (MAYV) is a related emerging alphavirus with epidemic potential with circulation overlap potential with CHIKV. We previously reported the ability of a non-replicating human adenovirus (AdV)-vectored vaccine expressing the MAYV structural polyprotein to protect against disease in mice following challenge with MAYV, CHIKV and UNAV. Herein, we evaluated mouse immunity and protective efficacy for an AdV-CHIKV full structural polyprotein vaccine in combination with heterologous AdV-MAYV prime/boost regimens versus vaccine coadministration. Heterologous prime/boost regimens skewed immunity toward the prime vaccine antigen but allowed for a boost of cross-neutralizing antibodies, while vaccine co-administration elicited robust, balanced responses capable of boosting. All immunization strategies protected against disease from homologous virus infection, but reciprocal protective immunity differences were revealed upon challenge with heterologous viruses. In vivo passive transfer experiments reproduced the inequity in reciprocal cross-protection after heterologous MAYV challenge. We detected in vitro antibody-dependent enhancement of MAYV replication, suggesting a potential mechanism for the lack of cross-protection. Our findings provide important insights into rational alphavirus vaccine design that may have important implications for the evolving alphavirus vaccine landscape.

## 1. Introduction

Chikungunya virus (CHIKV) is a mosquito-transmitted alphavirus that has caused numerous explosive epidemics since first emergence in 1952 and is actively causing outbreaks in Latin America, with over 320,000 cases reported so far in 2024 according to ECDC (as of 31 May). Mayaro virus (MAYV) is an emerging alphavirus first identified in 1954 that has caused outbreaks in Latin America and currently shares the potential for geographic cocirculation with CHIKV [1,2]. In contrast to CHIKV, MAYV has caused only small outbreaks; however, the disease burden and seroprevalence is likely vastly underestimated due to neutralization and ELISA diagnostic assay cross-reactivity with CHIKV and overlap in circulation with other arboviruses [3]. Antibody cross-reactivity between CHIKV and MAYV and other Semliki Forest antigenic complex alphaviruses has been observed in humans after infection [4,5,6] and vaccination [7,8]. Because of overlap in viral circulation, cross-reactive herd immunity is one hypothesis of why MAYV cases are not more common. The currently utilized mosquito vectors for CHIKV and MAYV differ, as do their suitable habitats, providing an additional hypothesis for the restricted range of MAYV distribution. Nevertheless, MAYV has the potential to opportunistically emerge outside of the Amazon basin where it is currently endemic because of competence in additional mosquito vectors as demonstrated experimentally [9,10,11]. Old World alphavirus infections are typically characterized by fever, rash, arthritis, myalgia, headache, and fatigue with rare infections manifesting eye pain and encephalitis. There are currently no approved countermeasures to combat these symptoms with the only treatment available being supportive care, although several antivirals [12,13] and monoclonal antibody therapies [14] are in pre-clinical development.

The landscape of CHIKV vaccines is rapidly evolving with the recent U.S. FDA, European Medicines Agency, and Health Canada approval of the live-attenuated IXCHIQ (VLA1553) vaccine, which is in ongoing clinical evaluations but was previously evaluated in mice [15,16], NHP [17,18], and in Phase I [19] and Phase III [20,21,22,23] human clinical trials. Several additional inactivated, mRNA [24], viral-vectored [25,26], and virus-like particle [27,28] CHIKV vaccines are in clinical development [29]. MAYV vaccine development is not as advanced, but approaches have included inactivated [30], live-attenuated [31,32,33], viral vectors [34,35], VLP [36], and DNA/RNA [37] platforms. Despite the approval of IXCHIQ, the development of additional alphavirus vaccines is warranted, as is research to understand the impacts of differential alphavirus exposure history on vaccine efficacy and how vaccine administration strategies can balance cross-reactive immunity.

The specific correlates of protection from alphavirus infection are still frequently debated, especially following approval of the first CHIKV vaccine. Neutralizing antibodies are the generally agreed upon dominant correlate, although the magnitude of the response necessary to achieve protection from disease and/or infection is still disputed and dependent upon the vaccine type, animal model, challenge dose, and assay variability and lack of standardization. For example, CHIKV 50% plaque reduction neutralization titers (PRNT_50_) as low as 10 [38] or 150 [17] and as high as an IgG titer of 10^4^ [15,16] have been proposed to be the correlate of protective immunity. While these thresholds have been determined from various mouse and NHP studies, there are conflicts in translation to other studies. Protection from alphavirus infection and pathogenesis relies on the cooperation of immune functions and protective roles for binding/neutralizing antibodies, CD8^+^ T cells, macrophages, and other immune responses [14,38,39,40]. One immune mechanism that challenges protection even in the presence of potent virus-specific antibodies is known as antibody-dependent enhancement (ADE), for which viral infectivity was first demonstrated in 1964 in chicken embryonic fibroblasts for replication of flaviviruses and the alphavirus, Getah virus (GETV) [41]. The ADE of disease in the presence of sub-neutralizing antibody levels has been well documented for human dengue virus (DENV) infection and is generally thought be a major clinical concern for flaviviruses only. ADE has been demonstrated for a diverse group of virus families including alphaviruses, which have implicated or hypothesized CHIKV [42,43] and Ross River virus (RRV) [44,45,46] in in vitro or mouse studies. Direct evidence of CHIKV infection enhancement in humans has not been reported to date but remains a topic warranting attention as to the impact it may have on disease outcomes. Two studies have provided encouraging evidence of the lack of risk of CHIKV-mediated ADE. One reported that pre-existing anti-CHIKV antibodies were shown to correlate with decreased symptomatic disease in a Philippine cohort [47]. Another study demonstrated that in the context of pre-existing CHIKV immunity, individuals immunized with CHIKV VLP (PXVX0317) did not display increased adverse events relative to baseline seronegative immunized individuals [48]. In contrast, a few vaccine studies in mouse models have observed enhanced viral replication or disease after CHIKV challenge and hypothesized that ADE was the responsible mechanism [15,16,49,50].

In light of the challenges associated with developing a protective alphavirus vaccine, especially in regions where multiple alphaviruses are endemic and/or reemerging causing the immune landscape to be in a general state of flux, we sought to evaluate different prime/boost vaccine regimens using a nonreplicating human adenovirus (AdV) vaccine platform to express the structural polyproteins of MAYV or CHIKV. We have previously shown that homologous prime/boost using the AdV-MAYV vaccine is protective against MAYV-, CHIKV-, and UNAV-induced disease in mice [34]. Herein, using both AdV-MAYV and a newly developed AdV-CHIKV construct, we aimed to evaluate heterologous prime/boost and coadministration vaccine strategies to assess MAYV or CHIKV homotypic disease and heterotypic cross-protection. We found that all vaccine administration strategies elicited homologous immunity with variable levels of cross-neutralization against heterologous viruses that correlated with the prime antigen. In addition, all of the vaccine strategies were capable of eliciting protection against footpad swelling disease but there were mixed cross-protective outcomes based upon quantification of viral dissemination in tissues. Passive transfer of immune sera from the various vaccine groups further demonstrated the lack of reciprocal cross-protection and showed that antibody-mediated immunity alone was insufficient to protect against disease, resulting in differential disease outcomes. Evaluation of antibody-dependent enhancement of infection in RAW264.7 cells demonstrated that diluted serum samples collected from each vaccine group had potential to enhance replication of both MAYV and UNAV but not CHIKV or RRV. Our findings shed light on potential differences in reciprocal cross-protective immunity for related alphaviruses, which may have implications on infection and vaccine coverage in populations with evolving alphavirus immunity.

## 2. Materials and Methods

### 2.1. Ethics Statement

Mouse experiments were performed at an ABSL-3 facility, accredited by the Association for Accreditation and Assessment of Laboratory Animal Care (AALAC) International, in accordance with the animal protocols approved by the Oregon Health and Science University (OHSU) Institutional Animal Care and Use Committee (IACUC Protocol #0913). Mice were housed in the ABSL-3 laboratory at the OHSU Vaccine and Gene Therapy Institute (VGTI) in ventilated racks with access to food and water with a 12 h light/dark cycle.

### 2.2. Cells, Viruses, and Viral Vaccine Vectors

Vero cells (ATCC CCL-81), 293IQ (Microbix, Mississauga, ON, Canada), and RAW264.7 cells (provided by Dr. Victor DeFilippis, OHSU, Beaverton, OR, USA) were propagated at 37 °C and 5% CO_2_ in Dulbecco’s Modified Eagle Medium (DMEM, Corning, Manassas, VA, USA) supplemented with 5% fetal bovine serum (FBS, Thermo Scientific, Waltham, MA, USA) and 1% penicillin–streptomycin-glutamine (PSG, Life Technologies, Carlsbad, CA, USA) (DMEM-5). *Aedes albopictus* C6/36 cells (ATCC CRL1660) were propagated at 28 °C with 5% CO_2_ in DMEM containing 10% FBS and 1X PSG.

Mayaro virus (MAYV_BeAr505411_, NR-49910), Una virus (UNAV_MAC150_, NR-49912), and Ross River virus (RRV_T-48_, NR-51457) were obtained though the Biodefense and Emerging Infectious Research Resources Repository (BEI Resources, Manassas, VA, USA). Chikungunya virus (CHIKV_181/25_) and CHIKV_SL15649_ were generated from infectious clones [40]. Alphaviruses were propagated in C6/36 cells by infection at MOI 0.1 and harvest of viral supernatant at 72 h post-infection (hpi). Supernatants were clarified and virus purified over a 10% sorbitol gradient by ultracentrifugation at 82,755× *g* for 70 min. Viral stocks were resuspended in 1X phosphate-buffered saline (PBS), aliquoted, and frozen for later use at −80 °C. Viral stocks were titered by limiting dilution plaque assays over confluent monolayers of Vero cells using a ten-fold dilution series from 10 to 10^−8^. Infected Vero cells were rocked for 2 h at 37 °C and overlaid with DMEM-5 containing 0.3% high/0.3% low viscosity carboxymethylcellulose (CMC, Sigma-Aldrich, St. Louis, MO, USA) (CMC-DMEM). Plaque assays for CHIKV_SL15649_, MAYV_BeAr505411_, UNA_MAC150_, and RRV_T48_ were fixed with 3.7% formaldehyde and stained with 0.2% methylene blue at 48 hpi. Plaque assays for CHIKV_181/25_ were fixed and stained at 72 hpi. Plaques were enumerated under a light microscope to determine viral stock titers. The limit of detection for plaque assays was 100 plaque forming units (PFU) per mL.

Adenovirus V (AdV) vaccine vectors were propagated in 293IQ cells. Construction of AdV-MAYV was previously described [34], and AdV-CHIKV was generated using similar techniques for the West African CHIKV_37997_ structural polyprotein sequence [34]. We chose CHIKV_37997_ as the vaccine strain because Akahata et al. showed that CHIKV_37997_ produces nearly 100-fold higher levels of virus-like particles compared to CHIKV_LR2006_ structural proteins, which we hypothesized to be an important feature for the generation of potent neutralizing antibody responses and subsequent protection [51].

### 2.3. Mouse Experiments

C57BL/6 mice (4–6 weeks old) were purchased from Jackson laboratories. Animals were immunized with 50 µL containing 10^8^ plaque forming units (PFU) of either AdV-MAYV and/or AdV-CHIKV diluted in 1X PBS injected in the left posterior thigh muscle. Animals were bled from saphenous veins at days 28 and 58 post-prime immunization for immunogenicity analysis. Serum was isolated from clotted blood following centrifugation for 5 min at 3000× *g*. For challenge experiments, female C57BL/6 mice were inoculated subcutaneously in the right footpad with 20 µL containing 10^4^ plaque forming units (PFU) of MAYV_BeAr505411_ or 10^3^ PFU of CHIKV_SL15649_. Footpad thickness height was measured daily after infection in the right (ipsilateral) rear footpad using a digital caliper according to the established method [52]. Mice were bled at 2 or 3 days post-infection (dpi) to quantify the level of serum viremia and animals were euthanized using isoflurane overdose at 5 or 7 dpi to characterize viral infection. Mouse ankle, quad, spleen, and heart tissues were collected in 1 mL of 1X PBS with approximately 250 µL of silica beads. Tissues were homogenized using a bead beater for three cycles of 45 s on and 30 s off. The tissue sample homogenates were centrifuged at 3000× *g* for 5 min to clear the debris. For the euthanized immunogenicity mice, splenocytes were isolated through a 70 µM cell strainer and washed with RPMI 1640 medium supplemented with 10% FBS and 1% PSG. Splenocytes were pelleted at 650× *g* for 10 min, and the red blood cells were lysed with 1X BioLegend red blood cell lysis buffer for 3 min, washed, and frozen for later use.

### 2.4. Neutralization Assays

Mouse serum was first heat inactivated by incubation for 30 min at 56 °C and then diluted by serial 2-fold dilutions in DMEM-5. The diluted sera were mixed with media containing approximately 70–120 plaque forming units of MAYV_BeAr505411_, CHIKV_181/25_, or UNAV_Mac150_ and incubated for 2 h at 37 °C with 5% CO_2_ with constant rocking. The mixtures were added to 12-well plates of confluent Vero cells and incubated for an additional 2 h at 37 °C with 5% CO_2_ with continuous rocking. CMC-DMEM-5 was then added to each well to overlay. Cells were incubated for 48 h for MAYV_BeAr505411_ and UNAV_Mac150_ or 72 h for CHIKV_181/25_. Plates were fixed and stained as described for the plaque assays above. Plaques were counted and the percent of plaques at each dilution relative to wells without serum were determined to calculate percent neutralization of infection. The 50% plaque reduction neutralization titers (PRNT_50_) were calculated by nonlinear regression analysis with variable slope using GraphPad Prism 9 software.

### 2.5. Viral RNA Detection

Total nucleic acids were isolated from 300 µL of each mouse tissue homogenate using the Promega Maxwell 48 sample RSC purification system with the Maxwell RSC Viral TNA extraction kit (Promega, Madison, WI, USA). Purified nucleic acids were resuspended in 70 µL of RNAse free water, and each sample was diluted to 100 ng/µL. ezDNase digestion was used to remove contaminating DNA. Single-stranded cDNA was generated from 1 µg of total RNA using random hexamers and reverse transcriptase Invitrogen Superscript IV, according to the manufacturer’s protocol. Gene amplicons were used as quantification standards. The primers and probe used to detect MAYV RNA were Forward-CCATGCCGTAACGATTGC, Reverse-CTTCCAGGCTGCCCGGCACCAT, and probe FAM-TGGACACCGTTCGATAC–MGB. The primers and probe used to detect CHIKV_SL15649_ RNA were Forward-CCGTCCCTTTCCTGCTTAGC, Reverse-AAAGGTTGCTGCTCGTTCCA, and Probe FAM-ACATACCAAGAGGCTGC. Quantitative RT-PCR was performed using a QuantStudio 7 Flex Real-Time PCR system in triplicate reactions. All data were analyzed using the Applied Biosystems QuantStudio 7 Flex Real-time PCR System software. The viral RNA levels were normalized to a murine housekeeping gene, ribosomal protein RPS17, and reported in copies per mL of tissue homogenate.

### 2.6. Quantification of Infectious Virus

Infectious virus in mouse serum, tissue homogenate, or RAW264.7 cell supernatants were quantified by limiting the dilution plaque assays, as described above. Briefly, 20 µL of serum, tissue homogenate, or RAW264.7 cell supernatants were added to 180 µL DMEM-5, which was serially diluted ten-fold. Viral dilutions were added to confluent monolayers of Vero cells plated in 48-well plates and allowed to incubate for 2 h at 37 °C with 5% CO_2_ with continuous rocking followed by addition of CMC-DMEM-5 overlay. Plaque assays were fixed and stained as described above.

### 2.7. ELISPOT

Splenocytes were added to mouse IFN-*γ* ELISPOT plates (Mabtech, Cincinnati, OH, USA) at a density of 2.5 × 10^5^ cells in RPMI with 5% FBS and 1% PSG and treated with the CHIKV E1 18-mer peptide #451 (CAVHSMTNAVTIREAEIE) [40] or MAYV E2 15-mer peptide #2 (LAKCPPGEVISVSFV) [34] at a final concentration of 10 µg/mL. A portion of the cells was stimulated with phorbol 12-myristate 13-acetate (PMA) at 25 ng/mL plus ionomycin at 500 ng/mL (positive control) or left untreated (negative control). Plates were wrapped in foil and incubated for 24 h at 37 °C with 5% CO_2_. Plates were washed once with 1X PBS and incubated with anti-mouse IFN-*γ* biotin antibody for 2 h. After a wash with 1X PBS, the plates were incubated with streptavidin-ALP antibody for 1 h. Spots were visualized following the addition of BCIP/NPT-plus substrate and washed a final time with 1X PBS before enumerating with an AID ELISPOT Reader Classic (AID, Penzberg, Germany). The results were background subtracted using the number of spots calculated in the negative control wells and the data are reported in spot forming units (SFU) per 1 million splenocytes.

### 2.8. Western Blot Analysis

Cell lysates were collected from 293IQ cells infected with AdV-MAYV or AdV-CHIKV at an MOI of 1. Lysates were loaded into 4–12% Bis/Tris polyacrylamide gels (ThermoFisher, Waltham, MA, USA), and the samples were electrophoresed for 45 min at 170 volts. Proteins were transferred to activated PVDF membranes for 25 min at 25 volts using a semi-dry electro-blotter. To detect MAYV proteins, membranes were probed for 1 h with serum diluted 1:500 from a mouse that was primed and boosted (30 days post-boost) with 10^8^ PFU of AdV-MAYV. To detect CHIKV proteins, membranes were probed for 1 h with serum diluted 1:700 from a mouse that was primed and boosted (30 days post-boost) with 10^8^ PFU of AdV-CHIKV. After extensive washing with TBS-Tween 20, the membranes were probed for 1 h with anti-mouse IgG secondary antibody (Rockland) diluted 1:10,000. Membranes were washed and developed with SuperSignal West Pico Plus chemiluminescent substrate solution (ThermoFisher) and exposed onto X-ray film.

### 2.9. Antibody-Dependent Enhancement Assays

RAW264.7 cells were seeded at a density of 10^5^ cells per well in a 96-well plate the day prior to the assay. Serum samples collected from vaccinated mice at 58 days post-prime were serially diluted 1:5 after an initial 1:10 dilution for 11 total dilutions in DMEM-5 and then mixed with 1 × 10^5^ PFU of MAYV_BeAr505411_, CHIKV_181/25_, UNAV_MAC150_, or RRV_T48_. A negative control well containing virus without serum was included as a baseline infection control. Media were removed from RAW264.7 cells and serum/virus dilutions were used to infect the cells by continuous rocking for 2 h at 37 °C with 5% CO_2_. After 2 h, infection media were removed and replaced with DMEM-5. At 24 h post-infection, viral supernatants were titered by limiting dilution plaque assay using Vero cells as described above. The fold increase in release of infectious virus was calculated relative to wells containing virus and no serum.

### 2.10. Statistical Analysis

All statistical analyses were performed using Graph Pad Prism 10 software. Nonlinear regressions with variable slopes were used to calculate neutralization titers. Neutralizing antibody titers among vaccine groups were compared by one-way ANOVA. Viral loads in mouse tissues, virus-specific T cells, and footpad swelling were compared by two-way ANOVA. Correlations between PRNT_50_ and dilution to maximum viral infection enhancement were compared by Spearman correlation. Peak fold viral infection enhancement data were compared by Kruskal–Wallis test.

## 3. Results

### 3.1. Adenovirus-Vectored Alphavirus Vaccines Elicit Virus-Specific Neutralizing Antibodies and T Cells in Mice

**Figure 1:**
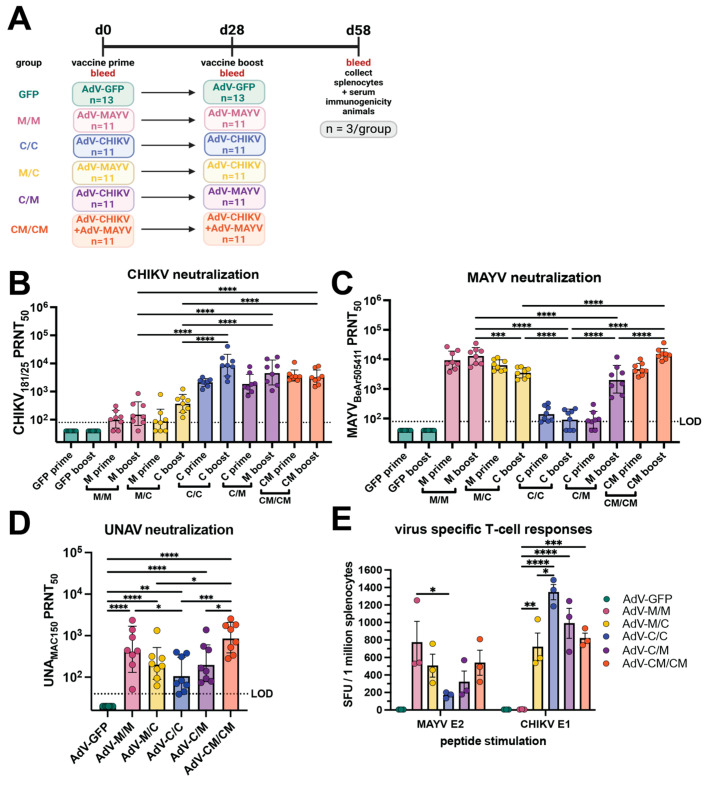
Immunogenicity of adenovirus-vectored alphavirus vaccines in C57BL/6 mice. (**A**) Study design schematic. C57BL/6 mice were immunized by intramuscular injections in the left posterior thigh muscle with human adenovirus V (AdV) vaccines expressing CHIKV or MAYV structural proteins at a dose of 10^8^ plaque forming units (PFU) each. Animals were boosted at day 28 with the homologous or heterologous vaccine or both in equivalent concentrations. At 58 days post-prime, three animals from each group were humanely euthanized for serum and splenocyte isolation. Sera from each mouse were collected at days 0, 28, and 58 post-prime for assessment of neutralizing activity against (**B**) CHIKV_181/25_, (**C**) MAYV_BeAr505411_, and (**D**) UNAV_MAC150_ using 50% plaque reduction neutralization tests (PRNT_50_) on confluent monolayers of Vero cells. In (**B**,**C**), the neutralization titer is shown at 28 days post-prime labeled as “prime” and 30 days post-boost labeled as “boost”. The neutralization titers were log-transformed, and the boosted (day 58 post-prime) titers are analyzed by one-way ANOVA with Holm-Šídák’s multiple comparisons. In (**D**), neutralization titers against UNAV for 8 mice in each group at day 58 post-prime are shown. The day 58 post-prime titers are analyzed by one-way ANOVA with Holm-Šídák’s multiple comparisons. For all neutralization data (**B**–**D**), the geometric means with geometric standard deviation are shown. The limit of detection (LOD) dilution titer for neutralization assays is 80 for CHIKV and MAYV and 40 for UNAV; samples with undetectable neutralizing activity are graphed as half of the LOD. In (**E**), splenocytes collected from each group of mice at day 58 post-prime were used to quantify virus-specific T cells against a MAYV E2 peptide and CHIKV E1 peptide using IFN-*γ* ELISPOT. The ELISPOT data reported as spot forming units per 1 million splenocytes were background subtracted from wells without peptide stimulation, and data were analyzed by two-way ANOVA with Tukey’s multiple comparisons; mean with SEM is shown. For (**B**,**C**,**E**), only select, significant comparisons are shown for simplicity with comparisons to AdV-GFP excluded. In all panels, biological replicates are plotted. For all statistical analyses, * *p* ≤ 0.05, ** *p* ≤ 0.01, *** *p* ≤ 0.001, and **** *p* ≤ 0.0001.

In previous studies, our group performed immunogenicity and protection studies in C57BL/6 mice using a nonreplicating human adenovirus-vectored (AdV) AdV-MAYV vaccine construct expressing the entire MAYV structural protein [34]. In mice, vaccination with AdV-MAYV is protective against disease elicited by infection with MAYV and partially protective against CHIKV and UNAV, suggesting that this vaccine elicits immunity against a range of serologically-related alphaviruses [34]. Herein, we sought to determine the following: (1) whether reciprocal protective immunity is elicited against MAYV and UNAV using an AdV-CHIKV vaccine that expresses the entire CHIKV_37997_ structural protein; and (2) whether cross-protective immunity could be improved by heterologous prime/boost or AdV co-administration strategies. To accomplish these goals, we vaccinated C57BL/6 mice with AdV-MAYV and/or AdV-CHIKV using homologous, heterologous, and coadministration immunization strategies and used vaccination with AdV-GFP as a vector control. Figure 1A depicts the vaccine strategy and bleeding schedule for this study. Mice were primed via intramuscular injection (i.m.) with 10^8^ plaque forming units (PFU) of AdV, bled, and then boosted at 28 days after receiving the priming vaccination. At 58 days post-prime, spleens and blood sera from three animals from each vaccine group were collected for the assessment of virus-specific T-cell responses and passive transfer experiments, respectively. Sera were collected from the remaining animals (*n* = 10 or 8, depending upon the group) for use in 50% plaque reduction neutralization tests (PRNT_50_) against CHIKV (Figure 1B), MAYV (Figure 1C), and UNAV (Figure 1D), with the results tabulated in Table 1. After homologous prime and boost regimens (C/C and M/M), the homotypic titers against their respective viruses were high but the titers against their heterotypic viruses were significantly lower, which demonstrates specificity against the homologous virus. Coadministration of AdV-CHIKV and AdV-MAYV (CM/CM) resulted in high serum neutralization dilution titers against all of the viruses including heterotypic UNAV. Neutralization titers for the heterologous prime boost groups (C/M and M/C) were significantly higher for the prime viral antigen and had limited boosting effect against CHIKV for the M/C group but titers against MAYV were boosted for the C/M group. Serum dilution titers against UNAV were similar for the heterologous prime boost groups. Together these findings suggest an interesting dichotomy in the responses generated by heterologous prime boost regimens and that the antibody responses are biased toward the priming vaccine antigen but can be overcome by vaccine co-administration. Prime and boost with only one AdV vaccine elicits limited cross-neutralization breadth. Additionally, we conducted an immunogenicity study in which a two-week rather than four-week interval between the prime and boost was assessed and little differences in antibody responses were observed; this experiment also validated that mice were mounting viral antigen-specific antibody responses (Supplemental Figure S1). Altogether, these data indicate that alphavirus AdV vaccine coadministration is a successful strategy for achieving balanced cross-neutralizing immunity against two pathogenic alphaviruses.

To examine T-cell-mediated vaccine-elicited immunity, virus-specific T-cells were quantified in splenocytes collected at day 58 post-prime (30 days post-boost) in IFN-*γ* ELISPOT assays against a MAYV E2 peptide and CHIKV E1 peptide. The M/M vaccine group generated a robust homotypic MAYV T-cell response at a mean of ~800 spot forming units (SFU) per million splenocytes, the highest MAYV-specific response of any vaccine group (Figure 1E). While MAYV T-cell responses in the M/C and CM/CM vaccine groups were not significantly lower than the M/M group (506 and 540 SFU, respectively), the numbers of MAYV specific T-cells in the C/M group was 321 SFU, and the heterotypic response for the C/C group was a mean of 169 SFU, which was significantly reduced (*p* = 0.0190) compared to the homotypic response of the M/M group (Figure 1E). For CHIKV-specific T-cells, the highest response was evoked in the C/C vaccine group at a mean of >1300 SFU (Figure 1E) followed by the C/M and CM/CM groups (990 and 820, respectively), which were not statistically different when compared to the C/C group (Figure 1E). The CHIKV T-cell response for the M/C group was significantly reduced compared to the homotypic response for the C/C group at a mean of 721 SFU (*p* = 0.0143) but a detectable cross-reactive T-cell response for the M/M group was not generated against the CHIKV E1 peptide (Figure 1E). These data indicated that cross-reactive virus-specific T-cells elicited by homologous vaccine administration strategies were limited and that cross-reactive responses could be achieved using heterologous and coadministration strategies. Together, these studies revealed limited cross-reactive alphavirus immunity elicited by the homologous vaccine administration strategies and supported that heterologous prime and boost or vaccine coadministration lead to greater antibody and T-cell breadth. Additionally, we observed a bias for the virus-specific antibody and T-cell immune response toward the priming vaccine antigen within the heterologous prime and boost groups.

### 3.2. All Vaccine Regimens Cross-Protect against MAYV and CHIKV-Induced Disease

**Figure 2:**
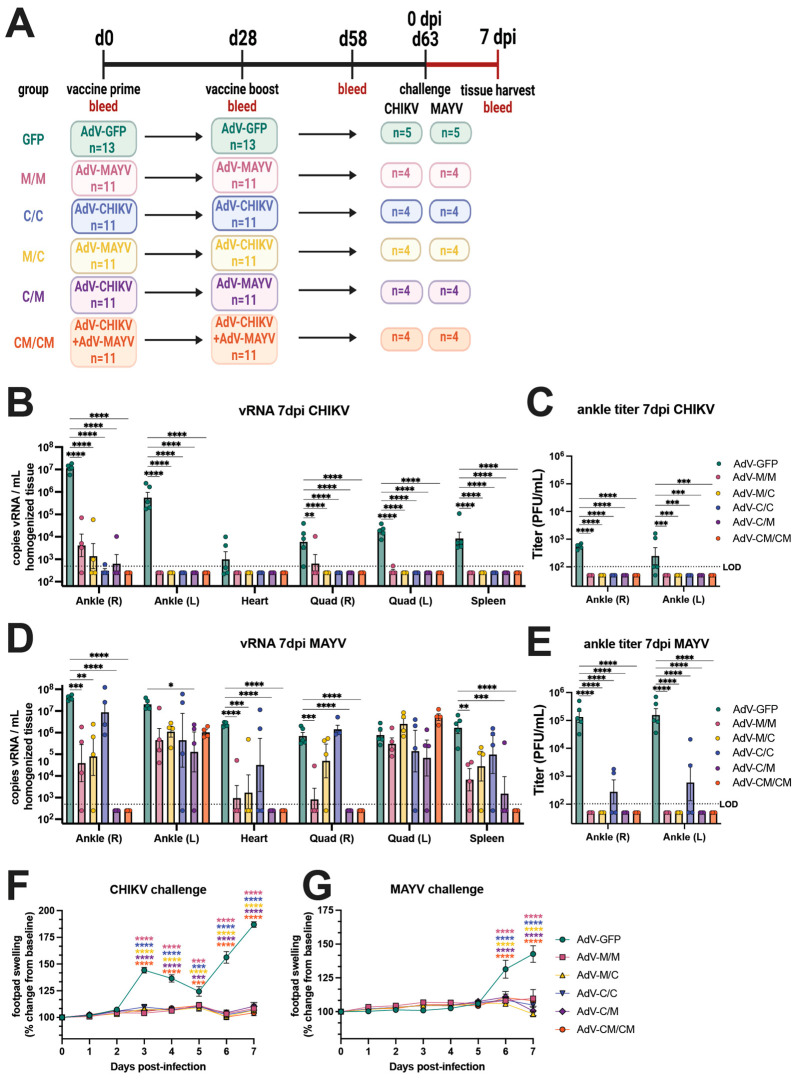
All CHIKV and MAYV vaccination strategies cross-protect against disease but this protection is not sterilizing. (**A**) Study immunogenicity and challenge efficacy study schematic. C57BL/6 mice were immunized with AdV vaccines by intramuscular injections in the left posterior thigh muscle. Animals were boosted at day 28 with the homologous or heterologous vaccine or both in equivalent concentrations. At 63 days post-prime, four animals per group were challenged by subcutaneous injection in the right footpad with 10^3^ PFU of CHIKV_SL15649_ or 10^4^ PFU of MAYV_BeAr505411_. Ankles, heart, quadriceps, and spleen tissues, as well as blood, were harvested from animals at 7 days post-infection. CHIKV viral RNA (vRNA) was quantified in mouse tissue homogenates by (**B**) qRT-PCR, and (**C**) infectious virus was quantified by limiting-dilution plaque assays. MAYV (**D**) vRNA levels and (**E**) infectious virus were also quantified in a similar manner. Infectious viral titers (**C**,**E**) are only shown for ankles as the quadriceps, heart, and spleen has no detectable titer at 7 days post-infection. Footpad swelling in the right (ipsilateral) rear footpad was measured daily after challenge for (**F**) CHIKV- and (**G**) MAYV-challenged mice. For all statistical analyses, two-way ANOVAs with Dunnett’s multiple comparisons were performed using log-transformed data. Only significant comparisons are shown where ns *p* > 0.05, * *p* ≤ 0.05, ** *p* ≤ 0.01, *** *p* ≤ 0.001, and **** *p* ≤ 0.0001. The limit of detection (LOD) for vRNA detection by qRT-PCR was 500 copies/mL of tissue homogenate and 100 PFU/mL for infectious viral plaque assays. For all graphs, means with SEM are plotted. In all panels, biological replicates are plotted.

To evaluate the ability of homologous, heterologous, and coadministration vaccine regimens to cross-protect against alphavirus infection and disease, we challenged the immunogenicity mice described in Figure 1 with 10^4^ PFU of MAYV_BeAr505411_ or 10^3^ PFU of CHIKV_SL15649_ by subcutaneous injection in the footpad at day 63 post-prime (day 33 postboost) (Figure 2A). Footpad swelling was measured daily and the mice were humanely euthanized at 7 days post-infection (dpi) for quantification of tissue viral burden using qRT-PCR and limiting dilution plaque assays. After CHIKV challenge, vRNA (Figure 2B) and infectious virus (Figure 2C) were nearly undetectable in the left (contralateral) ankle, heart, quadriceps, or spleen of all alphavirus immunized animals whereas AdV-GFP vaccinated controls had detectable CHIKV vRNA in all tissues that were tested (Figure 2B) and infectious virus in the ankles at 7 dpi (Figure 2C). The M/M and M/C vaccine groups had 10^3^–10^5^ copies/mL of CHIKV vRNA detected in the right ipsilateral ankle, and the C/C, C/M, and CM/CM groups had near sterilizing immunity with the exception of one mouse in the C/C group and one mouse in the C/M group (Figure 2B). Compared to the AdV-GFP control, all groups of alphavirus-vaccinated animals had significant reductions in CHIKV vRNA and infectious viral titers (Figure 2B,C). These results demonstrate that although the different vaccine groups had varying levels of CHIKV-neutralizing antibodies and virus-specific T cells (Figure 1B,E), the antiviral immunity was sufficient to significantly reduce or prevent viral dissemination to tissues after CHIKV challenge. In vaccinated mice that were challenged with MAYV, there were significant reductions in MAYV vRNA (Figure 2D) in the right (ipsilateral) ankle and heart compared to the AdV-GFP control group. There was also a significant inhibition in the production of infectious virus in the ankles (Figure 2E) for all alphavirus vaccine groups relative to AdV-GFP controls. In the right (ipsilateral) quadricep muscle and spleen, MAYV vRNA was significantly reduced compared to control mice for the M/M, C/M, and CM/CM vaccine groups (Figure 2D). However, the left (contralateral) ankle and left (contralateral) quadricep muscle were two sites where the viral burden was generally not significantly reduced relative to controls, although vRNA levels trended lower in the left (contralateral) ankle for all vaccine groups, except for the C/C group (Figure 2D). Despite the ability of the alphavirus vaccines to provide near-sterilizing protection against CHIKV infection, protection from MAYV infection was far from sterilizing, although significant reductions in MAYV vRNA in some tissues were observed and the infectious viral titers were inhibited, with the exception of the C/C group (Figure 2E). There was no infectious virus detected in any mouse in the quadriceps, heart, or spleen, even for the AdV-GFP-vaccinated controls; thus, these data were excluded from graphical presentation. While there were differential outcomes in the susceptibility of the alphavirus-vaccinated mice to CHIKV and MAYV infection as evidenced by viral tissue dissemination, the ability of all of the immunization strategies to protect against footpad swelling disease was striking. After CHIKV challenge, both homotypic and heterotypic protection from CHIKV-induced footpad swelling was observed for homologous, heterologous, and co-administration vaccine groups, which was statistically significant between 3 and 7 dpi (Figure 2F). Reciprocally after MAYV challenge, complete protection against MAYV-induced footpad swelling was observed for all alphavirus-vaccinated mice, which was statistically significant at 6 and 7 dpi (Figure 2G). These studies demonstrated that homologous, heterologous, and coadministration immunization strategies could greatly reduce viral burden after challenge, in some cases providing sterilizing immunity, and also cross-protect against inflammatory disease.

To further examine the differences in tissue viral burden in vaccinated animals, we performed a related experiment where animals were primed and boosted using the C/C, M/M, and CM/CM vaccination regimens, challenged at 61 days post-prime, and euthanized at 5 dpi rather than 7 dpi (Supplemental Figure S2A). Homologous prime boost resulted in high homotypic but low heterotypic antibody titers and, as similar to the previous experiment, vaccine coadministration resulted in high titers against both CHIKV and MAYV (Supplemental Figure S2B). At 2 dpi, serum MAYV viremia was significantly reduced for all alphavirus vaccine groups compared to the AdV-GFP control group (Supplemental Figure S2C). After CHIKV challenge, the infectious viral titers in the ankles revealed sterilizing protection elicited by the C/C and CM/CM vaccine groups but infectious virus was detected (~10^3^ PFU) in the right (ipsilateral) ankle for the M/M group (Supplemental Figure S2D). CHIKV vRNA levels were significantly reduced at 5 dpi in the spleen, quadriceps, ankles, and heart in the C/C and CM/CM vaccine groups; and the M/M vaccine group also showed reduced levels of vRNA, although this was not significant for all tissues (Supplemental Figure S2F). While a trend in reducing the viral burden after CHIKV infection at 5 dpi was observed, this was in contrast to near-sterilizing immunity observed at 7 dpi (Figure 2B). A similar antiviral outcome profile was observed after MAYV challenge, where little to no infectious virus was detected at 5 dpi for M/M and CM/CM vaccine groups, but four out of five mice in the C/C group had detectable infectious virus in the right ankle (Supplemental Figure S2E). When examining vRNA levels after MAYV infection, significant reductions in vRNA in the spleen, right quadricep muscle, ankles, and heart were observed for each vaccine group relative to the AdV-GFP controls (Supplemental Figure S2G). Comparable to the results observed in Figure 2D, the vaccine-elicited protection was not sterilizing against MAYV infection at 5 dpi (Supplemental Figure S2G).

### 3.3. Passive Antibody Transfer Demonstrates That Robust Cross-Neutralizing Antibody Responses Are Not Sufficient to Provide Sterilizing Cross-Protection against Infection or Disease

**Figure 3:**
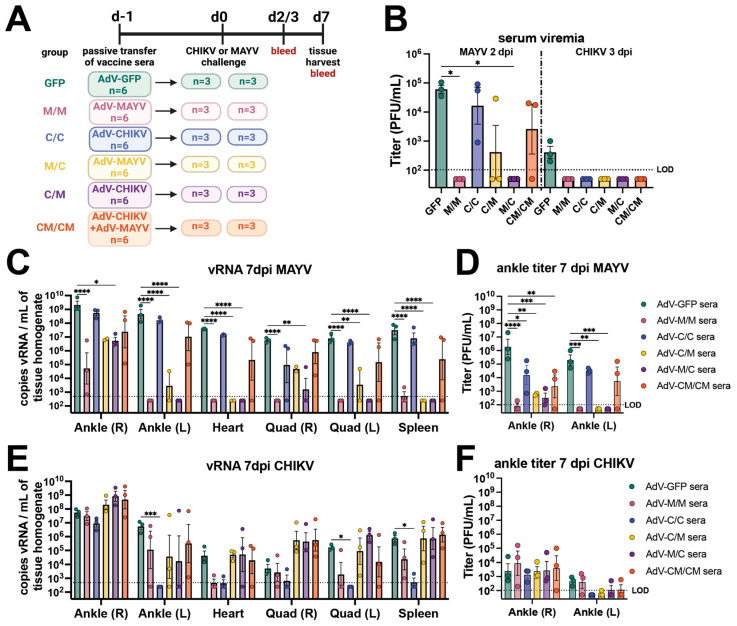
Passive transfer of vaccine immune sera demonstrates that antibodies are not sufficient for sterilizing cross-protection against viral replication in tissues. (**A**) Study schematic. Serum collected from three mice from each vaccine group described in Figure 1 was pooled and utilized for passive transfer by 100 µL i.p. injection in each of six mice. The next day, three mice were challenged by subcutaneous right footpad injection with 10^4^ PFU of MAYV_BeAr505411_, and three mice were challenged with 10^3^ PFU of CHIKV_SL15649_. MAYV-infected animals were bled at 2 days post-infection (dpi) and 3 dpi following CHIKV infection. Limiting dilution titering was used to measure serum viremia and infectious viral loads in the ankle, quadriceps, heart, and spleen tissues collected at 7 dpi. (**B**) Serum titers quantified by limiting-dilution plaque assays measured in PFU/mL. (**C**) MAYV vRNA quantified by qRT-PCR in tissues and (**D**) MAYV infectious viral titers in ankles quantified by plaque assay at 7 dpi. (**E**) CHIKV vRNA quantified by qRT-PCR in tissues and (**F**) CHIKV infectious viral titers in ankles quantified by plaque assay at 7 dpi. All values are log-transformed in (**B**–**F**). Serum titers (**B**) are analyzed with a Kruskal-Wallis test with Dunn’s multiple comparisons. Tissue titers and vRNA levels reported in (**C**–**F**) are analyzed by two-way ANOVA with the Dunnett’s multiple comparisons test. For simplicity in all graphs, only comparisons of *p* < 0.05 are shown where * *p* ≤ 0.05, ** *p* ≤ 0.01, *** *p* ≤ 0.001, and **** *p* ≤ 0.0001. For all graphs, mean with SEM are plotted. The limit of detection (LOD) for all plaque assays was 100 PFU/mL of serum or tissue homogenate with undetectable samples graphed as half of the LOD. The LOD for qRT-PCR assays was 500 vRNA copies/mL of tissue homogenate.

To investigate the impact that antibodies have on the nonreciprocal vaccine-induced cross-protection following viral challenge, passive transfer experiments using stored serum from the immunogenicity mice (*n* = 3/group; 58 days post-prime) for each of the five vaccine groups and AdV-GFP control were conducted (presented in Figure 1). One day before viral challenge with 10^3^ PFU of CHIKV_SL15649_ or 10^4^ PFU of MAYV_BeAr505411_, 100 µL of mouse vaccine immune serum was administered via intraperitoneal (i.p.) injection to naïve C57BL/6 mice (Figure 3A). Passive transfer of sera from the M/M and M/C vaccine groups protected against MAYV viremia (*p* ≤ 0.05), whereas two out of three mice in the C/M serum transfer group did not have detectable viremia (Figure 3B). In contrast, C/C sera provided little cross-protection against MAYV serum viremia with two of three mice developing similar serum viremia (mean ~5 × 10^4^ PFU/mL) to AdV-GFP passive transfer controls and the third mouse having a serum viremia equal to 9 × 10^2^ PFU/mL (Figure 3B). For passive transfer of CM/CM vaccine sera (the vaccine group with similar MAYV-neutralizing antibody potency as the M/M homologous group as shown in Figure 1C), two of three animals developed serum viremia (~2 × 10^4^ PFU/mL) that was slightly reduced relative to AdV-GFP control animals (Figure 3B). A similar trend in tissue distribution was observed for both MAYV vRNA (Figure 3C) and infectious virus (Figure 3D), as the C/C and CM/CM serum passive transfer groups did not show a reduction in viral loads and were similar to AdV-GFP control animals. The M/M, C/M, and M/C serum passive transfer groups had significant reductions in MAYV vRNA in the left (contralateral) ankle, quadricep muscles, spleen, and heart (Figure 3C) as well as infectious viral titers in the ankles (Figure 3D). Viral burden across all tissues was similar in magnitude for the C/C and CM/CM serum passive transfer groups to the infected AdV-GFP control animals (Figure 3C,D). This revealed that antibody from M/M, M/C, or C/M vaccine administration strategies could substantially block MAYV replication in tissues, but that homologous C/C and co-administration CM/CM vaccine strategies resulted in antibody responses that had limited impacts on reducing MAYV viral dissemination. Together, these data indicate that robust neutralizing antibody potency alone in this context does not translate to complete protection from MAYV infection.

At 3 days post-CHIKV infection, all alphavirus vaccine passive transfer groups prevented serum viremia (Figure 3B). The homotypic C/C serum passive transfer group provided near-sterilizing protection (Figure 3E,F) with the exception of low amounts of vRNA detected in heart, quadricep muscles, and spleen subsets and at the right (ipsilateral) ankle challenge injection site. Although the other vaccine administration strategies (C/M, M/M, M/C and CM/CM) elicited near sterilizing protection from CHIKV infection (Figure 2B,C,F), passive transfer experiments of sera from these groups were not sufficient to significantly reduce viral loads, with several mice actually displaying increased CHIKV vRNA levels in multiple tissues compared to those animals receiving the AdV-GFP control sera (Figure 3E).

**Figure 4:**
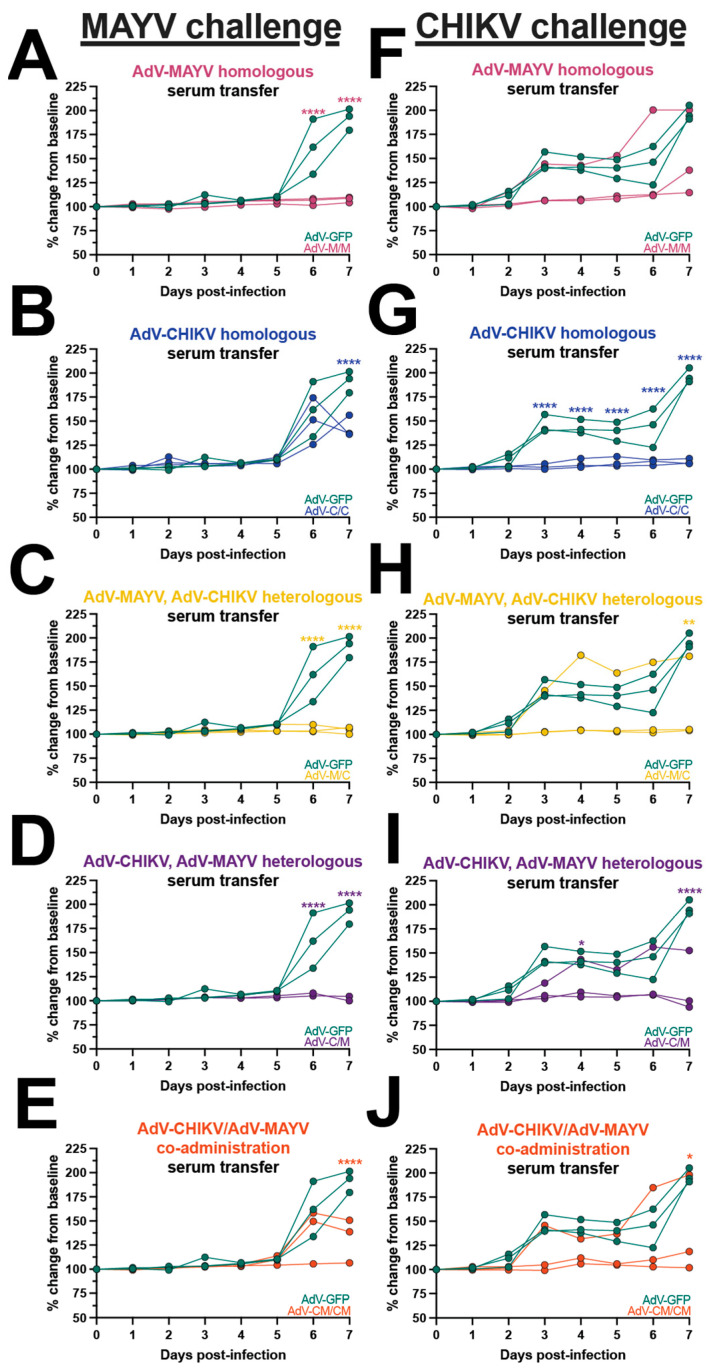
Differential disease outcomes elicited by passive antibody transfer. Naïve mice received mouse vaccine sera via passive antibody transfer and were challenged by subcutaneous injection into the footpad with CHIKV or MAYV. Footpad swelling was measured daily with digital calipers and mice were humanely euthanized at 7 days post-infection (dpi). Footpad swelling after MAYV challenge is plotted adjacent to control animals who received AdV-GFP sera or (**A**) AdV-MAYV homologous sera, (**B**) AdV-CHIKV homologous sera, (**C**) AdV-MAYV, AdV-CHIKV heterologous sera, (**D**) AdV-CHIKV, AdV-MAYV heterologous sera, or (**E**) AdV-CHIKV/AdV-MAYV co-administration sera transfer. Footpad swelling after CHIKV challenge is plotted adjacent to animals who received AdV-GFP sera for (**F**) AdV-MAYV homologous sera, (**G**) AdV-CHIKV homologous sera, (**H**) AdV-MAYV, AdV-CHIKV heterologous sera, (**I**) AdV-CHIKV, AdV-MAYV heterologous sera, and (**J**) AdV-CHIKV/AdV-MAYV co-administration sera transfer. Raw measurements were used to calculate percent change from baseline which is plotted for individual mice. These values were compared to AdV-GFP serum-transferred controls and analyzed using a two-way ANOVA with the Šídák’s multiple comparisons test where * *p* ≤ 0.05, ** *p* ≤ 0.01, and **** *p* ≤ 0.0001.

Footpad swelling outcomes for animals in the passive transfer experiments following infection with either MAYV or CHIKV are shown in Figure 4. All of the MAYV challenge mice receiving AdV-GFP sera developed footpad swelling peaking at 7 dpi (Figure 4). Consistent with our analysis of MAYV-infected tissues, mice receiving sera from the M/M, M/C, and C/M vaccine groups were significantly protected from MAYV-induced footpad swelling (Figure 4A,C,D). In contrast, MAYV-induced footpad swelling was observed in mice receiving the serum from the C/C and CM/CM vaccine groups (Figure 4B,E). Interestingly, the mice in the C/C serum passive transfer group developed footpad swelling that was similar in magnitude to the AdV-GFP controls at 6 dpi, but the level was reduced at 7 dpi (*p* ≤ 0.0001; compared to AdV-GFP) (Figure 4B). A similar footpad swelling phenotype was observed for the CM/CM serum passive transfer mice, with a reduction at 7 dpi (*p* ≤ 0.0001; compared to AdV-GFP) (Figure 4E). Footpad swelling following CHIKV infection was reduced in animals receiving serum from the M/M, M/C, C/M, and CM/CM groups; however, for each group, two mice were fully protected and one mouse developed footpad swelling with similar kinetics to control mice (Figure 4F,H–J). The C/C serum passive transfer group was completely protected from footpad swelling, reaching statistical significance (*p* ≤ 0.0001) (Figure 4G). These outcomes provide examples of differential disease outcomes in instances of varying levels of cross-neutralizing antibody potency based upon the vaccine antigen delivery regimen. These findings support observations of nonreciprocity in cross-protection for CHIKV and MAYV infection and suggested that other immune responses may also contribute to cross-protection.

### 3.4. Serum from Immunized Mice Exhibits Antibody-Dependent Enhancement Activity of MAYV and UNAV Replication In Vitro

**Figure 5:**
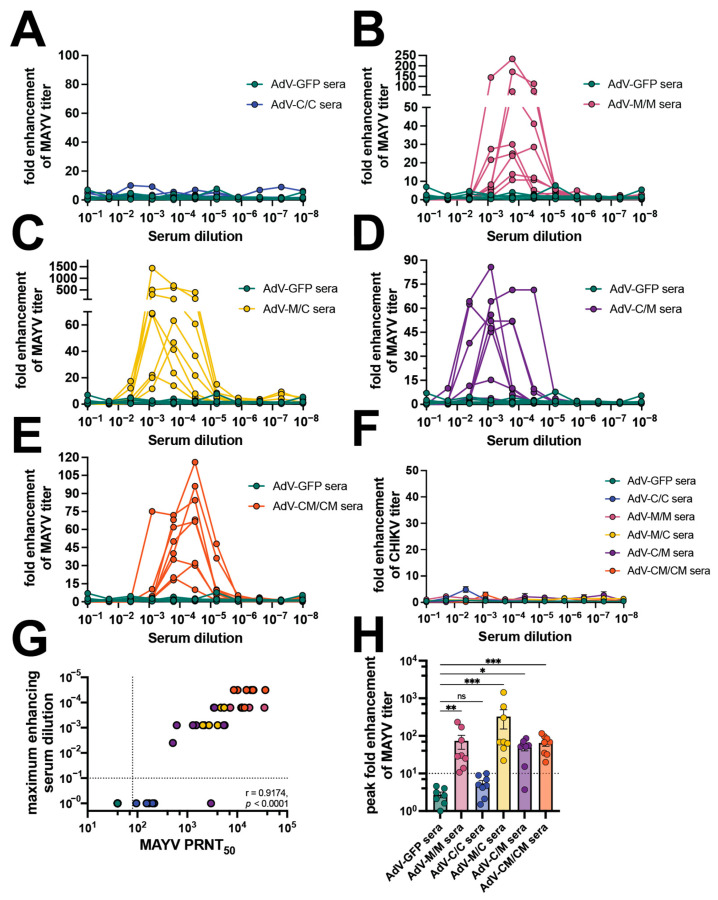
Vaccine sera enhance MAYV but not CHIKV replication in mouse macrophages. Mouse sera collected at 58 days post-prime, as described in Figure 1, were used in antibody-dependent enhancement (ADE) of infection assays in which 10^5^ RAW264.7 cells per well were infected with 1:5 serial dilutions of mouse sera mixed with an MOI 1 of (**A**–**E**) MAYV_BeAr505411_ or (**F**) CHIKV_181/25_. Cells were incubated with infection mixtures for 2 h, media were replaced, and cells were incubated for 20–24 h at 37 °C. Viral supernatants from the RAW cells were collected and titered by limiting-dilution plaque assays. Fold enhancement of viral titer was calculated relative to wells infected with virus only without serum. ADE assays were performed for (**A**) AdV-C/C sera, (**B**) AdV-M/M sera, (**C**) AdV-M/C sera, (**D**) AdV-C/M sera, and (**E**) AdV-CM/CM sera all compared to AdV-GFP control sera. (**G**) Spearman’s correlation of 58 days post-prime MAYV PRNT_50_ (reported in Figure 1) versus maximum MAYV-enhancing serum dilution in log scale. (**H**) Compilation of peak fold enhancement of MAYV titer values for each vaccine serum group analyzed by Kruskal–Wallis test with Dunn’s multiple comparisons where ns *p* > 0.05, * *p* ≤ 0.05, ** *p* ≤ 0.01, and *** *p* ≤ 0.001. All ADE assays were performed using 7 AdV-GFP and 8 AdV vaccine serum biological replicates for MAYV ADE assays or 4–5 AdV vaccine serum biological replicates for CHIKV assays. For (**F**,**H**), error bars are SEM. Supplemental Figure S3 contains the raw titer values used to calculate fold enhancement of MAYV and CHIKV titer.

There are only a limited number of reports of antibody-dependent enhancement (ADE) of alphavirus infection but none, to date, have evaluated infection enhancement across different virus species. Since the vaccination and passive transfer experiments demonstrated an inequity in reciprocal cross-protection after heterologous MAYV challenge, we interrogated the in vitro ADE potential in macrophages for the adenovirus-vectored alphavirus vaccine-elicited antibodies. Using sera from each vaccine group collected from immunized mice shown in Figure 1, we first established ADE assays in which sera were serially diluted with MAYV or CHIKV and used to infect RAW264.7 cells (Figure 5, Supplemental Figure S3). Viral supernatants were collected at 24 h post-infection and titered by limiting dilution plaque assays. The fold enhancement of viral infection was calculated relative to wells of infected cells without sera and enhancement was defined as a ≥10-fold increase in viral titer released from cells that were infected in the presence of diluted sera. Sera from AdV-GFP control vaccinated animals did not cause infection enhancement at any dilution. Similarly, dilution of the C/C sera did not lead to enhancement of MAYV infection (Figure 5A), which we hypothesized was also due to low levels of cross-neutralizing antibody titers with a GMT = 95 (Table 1). However, in the presence of M/M, M/C, C/M, and CM/CM diluted sera, increased infection of MAYV was observed for nearly all of the sera evaluated with the exception of one sample in the C/M group (Figure 5B–E). The serum dilution to peak enhancement of MAYV replication varied between each vaccine group with the M/C group having the lowest dilution range to peak ADE of 1:1250 to 1:6250 (Figure 5C), while the M/M, C/M, and CM/CM groups (Figure 5B,D,E) had peak enhancing-serum dilutions out to 1:31,250 (Table 1). In contrast, none of the serum samples from any vaccine group led to enhancement of CHIKV replication (Figure 5F). The MAYV-neutralizing titer values directly correlated with the maximum enhancing-serum dilution (*p* < 0.0001) (Figure 5G). The peak fold enhancement of MAYV titer for the M/C group was statistically significant relative to control wells at up to 1437-fold and was slightly reduced for the M/M, C/M, and CM/CM groups but ranged up to 233-, 86-, and 116-fold, respectively (Figure 5H, Table 1). These results demonstrated a range of vaccine-elicited neutralizing antibody potency that led to the enhancement of MAYV but not CHIKV replication in mouse macrophage cells. These findings also revealed the ability of both homotypic and heterotypic alphavirus-neutralizing antibodies to cause ADE in mouse macrophages.

**Figure 6:**
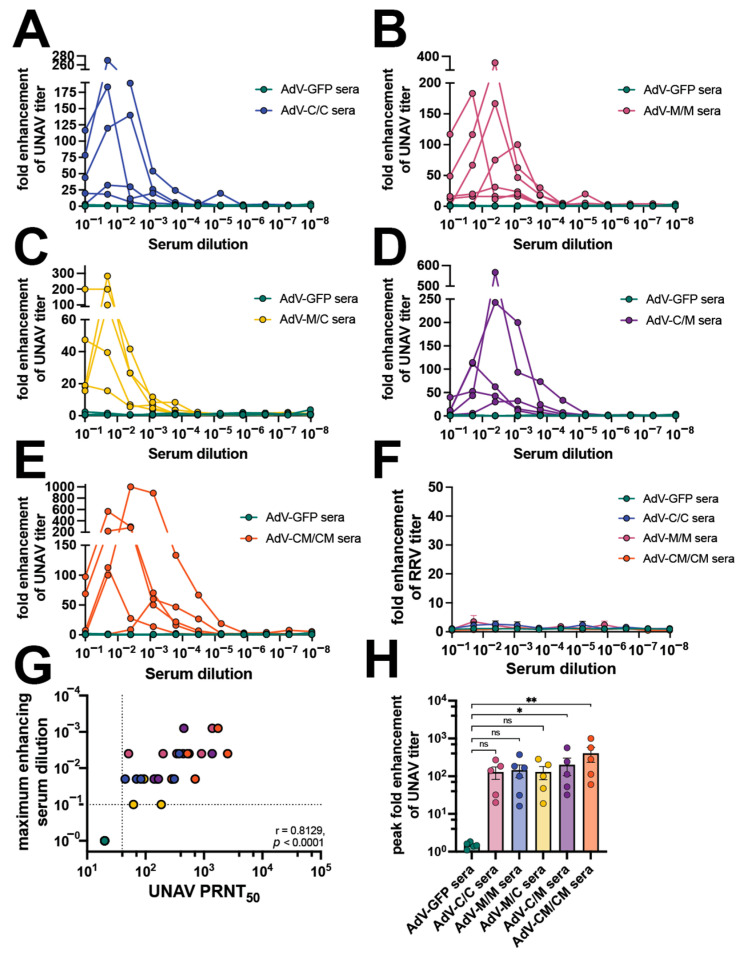
Vaccine sera enhance UNAV replication in mouse macrophages. Mouse sera collected at 58 days post-prime (as described in Figure 1) were used in ADE of infection assays in which 10^5^ RAW264.7 cells per well were infected with 1:5 serial dilutions of mouse sera mixed with an MOI 1 of (**A**–**E**) UNA_MAC150_ or (**F**) RRV_T48_. Cells were incubated with infection mixtures for 2 h, media were replaced, and cells were incubated for 20–24 h at 37 °C. Viral supernatants from the RAW cells were collected and titered by limiting-dilution plaque assays. Fold enhancement of viral titer was determined relative to wells infected with virus only without serum. ADE assays were performed for (**A**) AdV-C/C sera, (**B**) AdV-M/M sera, (**C**) AdV-M/C sera, (**D**) AdV-C/M sera, and (**E**) AdV-CM/CM sera all compared to AdV-GFP control sera. (**G**) Spearman’s correlation of 58 days post-prime UNAV PRNT_50_ (reported in Figure 1) versus maximum UNAV-enhancing serum dilution in log scale. (**H**) Compilation of peak fold enhancement of UNAV titer values for each vaccine serum group analyzed by Kruskal–Wallis test with Dunn’s multiple comparisons where ns *p* > 0.05, * *p* ≤ 0.05, ** *p* ≤ 0.01. All assays were performed using 5 AdV-GFP and 5–6 AdV vaccine serum as biological replicates. Supplemental Figure S4 contains the raw titer values used to calculate fold enhancement of UNAV and RRV titer.

Antibody-mediated enhanced infection assays were extended to additional alphaviruses (UNAV and RRV) in RAW264.7 cells (Figure 6, Supplemental Figure S4). In this case, where a higher potency of UNAV cross-neutralizing antibodies was present in the C/C group (Table 1), we observed enhancement of UNAV replication in the presence of diluted sera, reaching between 20- and 270-fold enhancement of viral titers for serum dilutions ranging 1:10 to 1:250 (Figure 6A, Table 1). Diluted vaccine sera from each of the tested samples in the M/M, M/C, C/M, and CM/CM groups also enhanced UNAV infection in macrophages (Figure 6B–E, Table 1). The serum dilution to peak UNAV enhancement ranged from 1:10 dilution to 1:1250 for M/M, M/C, C/M, and CM/CM vaccine groups (Figure 6B–E, Table 1). We did not detect any substantial ADE activity for RRV using any of the serum samples (Figure 6F). For the UNAV ADE, we found that the UNAV PRNT_50_ correlated with the dilution to maximum enhancement (*p* < 0.001) (Figure 6G). The peak fold enhancement of viral titer was statistically significant compared to AdV-GFP sera, ranging 16-1000-fold (Figure 6H, Table 1). These experiments demonstrate the ability of heterotypic alphavirus-neutralizing antibodies to cause ADE of UNAV and MAYV in mouse macrophages, interestingly these two viruses are closely related, which may be important for their similar results but further studies will be required to fully understand this outcome.

## 4. Discussion

The ongoing CHIKV outbreak occurring in Latin America where other related alphaviruses are known to circulate has warranted studies to assess the cross-protective dynamics of prior infection or immunity imparted by vaccination. In this study, we evaluated immunization strategies in immunocompetent mice using our previously reported nonreplicating human adenovirus-vectored (AdV) vaccine expressing the MAYV structural polyprotein and a similar AdV-CHIKV vaccine expressing the CHIKV structural polyprotein. We compared both the immunogenicity and protective capacity of these vaccines in C57BL/6 mice in homologous, heterologous and co-administration prime and boost strategies. Overall, our findings revealed that heterologous and coadministration immunization strategies are effective to achieve cross-reactive immunity but incompletely equate to balanced cross-protection. These observations have significance for multivalent alphavirus vaccine design and administration.

One of our major findings was nonreciprocity in CHIKV versus MAYV cross-protection. Passive transfer experiments revealed that antibody potency and other immune responses may have differential contributions to the threshold of protection against MAYV and CHIKV infection. One study demonstrated CHIKV infection elicited immunity can protect against MAYV infection and vice versa, but that vaccine-elicited cross-protective immunity is more complicated and harder to achieve [53]. Adenovirus-vectored vaccines have been previously developed for CHIKV [26,35,54,55,56,57] and for MAYV by our group [34] and others [35]. The partial cross-protection against MAYV afforded by CHIKV-specific vaccines has been reported using an adenovirus-vectored vaccine [35] and live-attenuated or chimeric vaccines [33]; our study corroborates these findings and shows how heterologous and coadministration immunization strategies are insufficient to fully prevent infection but can provide protection against disease. Consistent with our study, greater protection from infection was observed in context of MAYV immunization and CHIKV challenge compared to the reciprocal CHIKV vaccine with MAYV challenge [35]. In our previous study, we demonstrated near-sterilizing protection elicited by AdV-MAYV against lethal CHIKV and UNAV challenge in IFN⍺R1^−/−^ mice, a very stringent model [34]. The differential phenotypes in cross-protection observed in the literature also underscore the importance of utilizing a range of animal disease models to evaluate vaccine efficacy. Future studies should investigate the vaccine administration strategies presented in this manuscript in lethal challenge models of immunodeficiency to more definitively identify the necessary and sufficient immune players in cross-protection. Overall, our findings, as well as these two published studies, continue to suggest differences in CHIKV and MAYV reciprocal cross-protection [33,35].

We also observed nonreciprocity in ADE assays with little to no enhancement for CHIKV or RRV but robust activity for MAYV and UNAV. Previous studies have shown that MAYV-neutralizing antibodies require Fc effector functions to be protective [58] and that nonneutralizing antibodies can also confer protection from alphavirus infection mediated by Fc effector functions and monocytes [59]. Especially given the range of neutralizing antibody potency resulting in ADE, a possibility is that the Fc effector functions elicited by the adenovirus-vectored vaccines in our study were not equal within or across vaccine groups, explaining both the partial protection phenotypes after passive transfer and range of the magnitude of ADE we observed. Although characterization of Fc effector functions was outside the scope of the current study, correlates of vaccine-elicited Fc effector functions and protection from infection and disease is an area that warrants further investigation. The lack of evidence of in vitro ADE in mouse macrophages for CHIKV in our study remains an enigma. CHIKV replication is reduced in macrophages compared to other cell types, however, CHIKV is known to persist in activated macrophages [60] and infected macrophages are a source of arthritogenic inflammatory cytokines [61,62]. The mechanisms of macrophage persistence of other alphaviruses like MAYV and UNAV have not been well characterized and may have some contribution to the virus-specific ADE phenotype we observed. Important limitations of our work for future consideration are the establishment of ADE assays in additional cell types, examination of viral RNA levels, and examination of viral output at later timepoints due to CHIKV persistence in macrophages. Our results here do not mean to exclude the possibility of CHIKV in vitro ADE, but rather to present an observed phenotype for MAYV and UNAV that was not recapitulated for CHIKV, which warrants further characterization. In vitro ADE studies of alphavirus infection to elucidate cell- and virus-specific differences, as well as studies that examine translation of the findings in vivo, are areas that warrant further research.

Another major finding from our study was that the boosting of antibodies by heterologous vaccine regimens was more limited compared to coadministration or homologous boosting. Original antigenic sin is known to limit boosting to antigenic determinants that were recognized during the priming event and may be limiting increased breadth of our heterologous boosting regimen [63]. We and others have previously determined that cross-reactivity between human CHIKV antisera for recognition of MAYV, in large part, is driven by responses against the E2 B domain [4,5,8,64,65,66,67,68,69]. The similarity between these two viruses may focus immunity against the common epitopes found in this or other similar regions. Future studies should further characterize vaccine-elicited antibodies beyond binding and neutralizing functions to see whether other antibody characteristics correlate with vaccine-elicited protection.

Our results from this study suggest important considerations for multivalent vaccine design and heterologous immunization strategies. Our conclusions highlight the need to characterize immunity and disease responses in those that have been infected with different but related alphaviruses, as the ongoing CHIKV outbreak in Latin America continues to affect a large number of individuals that are also in regions susceptible to MAYV or other alphaviruses. Type-specific, balanced immune responses are very important for DENV vaccine efficacy and safety due to risk of enhanced disease mediated by ADE. Our findings suggest that while heterologous and coadministration strategies can achieve balanced cross-reactive immunity, these responses do not translate to complete protection from infection or disease, and these antibodies also have in vitro ADE potential that may or may not be translational in vivo. Balance of alphavirus cross-reactive immunity and translation to achieve protection without risk of ADE is a concept that should be carefully considered when developing alphavirus vaccines.

## Figures and Tables

**Table 1:** Summary of alphavirus-neutralizing antibody titer responses (*n* = 8) and in vitro antibody-dependent enhancement activity in RAW264.7 at 58 days post-prime.

	**CHIKV_181/25_**	**MAYV_BeAr505411_**	**UNA_MAC150_**
AdV-C/C			
Min–Max PRNT_50_	2257–40,820	40–217	40–358
GMT	8690	95	116
Mean PRNT_50_	12,354	119	174
PRNT_50_ standard deviation	12,280	75	159
Observed ADE (≥10-fold)	0/4	0/8	5/5
Serum dilution to maximum ADE	NE	NE	1:10–250
Maximum fold enhancement of viral titer	NE	NE	20–270
AdV-M/M			
Min–Max PRNT_50_	40–849	5413–35,145	51–2349
GMT	162	13,115	460
Mean PRNT_50_	250	15,658	764
PRNT_50_ standard deviation	268	9877	769
Observed ADE (≥10-fold)	0/4	8/8	6/6
Serum dilution to maximum ADE	NE	1:6250–31,250	1:50–1250
Maximum fold enhancement of viral titer	NE	10.7–233	16–372
AdV-M/C			
Min–Max PRNT_50_	119–1235	2043–5597	62–1333
GMT	377	3526	214
Mean PRNT_50_	474	3768	331
PRNT_50_ standard deviation	352	1413	414
Observed ADE (≥10-fold)	0/5	8/8	5/5
Serum dilution to maximum ADE	NE	1:1250–6250	1:10–50
Maximum fold enhancement of viral titer	NE	22–1437	19–200
AdV-C/M			
Min–Max PRNT_50_	1076–16,927	512–12,048	74–1383
GMT	4733	2124	214
Mean PRNT_50_	7008	3481	368
PRNT_50_ standard deviation	5970	3837	449
Observed ADE (≥10-fold)	0/5	7/8	5/5
Serum dilution to maximum ADE	NE	1:250–31,250	1:50–1250
Maximum fold enhancement of viral titer	NE	15–86	32–567
AdV-CM/CM			
Min–Max PRNT_50_	1491–8074	8568–36,537	282–2540
GMT	3231	15,154	892
Mean PRNT_50_	3812	16,680	1171
PRNT_50_ standard deviation	2458	8812	828
Observed ADE (≥10-fold)	0/4	8/8	5/5
Serum dilution to maximum ADE	NE	1:6250–31,250	1:50–1250
Maximum fold enhancement of viral titer	NE	20–116	60–1000

Geometric mean titer = GMT; not enhanced = NE; enhancement is defined as a ≥10-fold increase in viral titer.

## Data Availability

The raw data supporting the conclusions of this article will be made available by the authors on request, please contact the corresponding author.

## Supplementary Materials

The following supporting information can be downloaded at: https://www.mdpi.com/article/10.3390/vaccines12090970/s1, Figure S1: Preliminary immunogenicity analysis; Figure S2: Vaccine cross-protection at 5 dpi; Figure S3: Raw titer data for MAYV and CHIKV ADE assays; Figure S4: Raw titer data for UNAV and RRV ADE assays.
