# Supplementary material for: Nonreciprocity in CHIKV and MAYV Vaccine-Elicited Protection
Source: Vaccines (Basel). 2024 Aug 27;12(9):970. doi: 10.3390/vaccines12090970 (PMC11435824; doi:10.3390/vaccines12090970)
Supplement: Supplementary file 1 [file vaccines-12-00970-s001.zip › vaccines-3140505-supplementary.pdf]

Supplemental figures

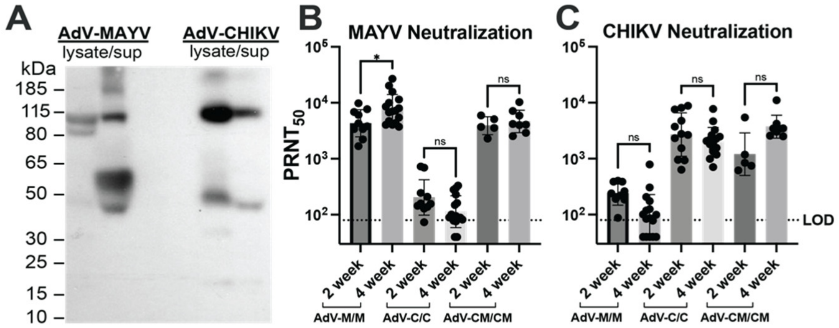

Supplemental Figure S1. Preliminary immunogenicity analysis. In preliminary mouse studies, the virus-specific immune response was validated for the previously tested AdV-MAYV and new AdV-CHIKV vaccine constructs. (**A**) Confirmation of structural polyprotein expression within the supernatant and lysate of 293IQ cells by western blot. AdV-MAYV was probed with primary mouse sera after AdV-MAYV homologous prime and boost (10^8 PFU i.m./dose) and AdV-CHIKV was probed with primary mouse sera after AdV-CHIKV homologous prime and boost (10^8 PFU i.m./dose). Neutralizing activity was assessed using 50% plaque reduction neutralization tests ($PRNT_{50}$) with Vero cells against (**B**) MAYV and (**C**) CHIKV. Neutralizing antibody titers at two and four weeks post-prime were compared using one-way ANOVA with Holm-Šídák's multiple comparisons where *P = 0.0113 and ns $P > 0.05$. The limit of detection for neutralization assays is 80. Bars are mean with SEM.

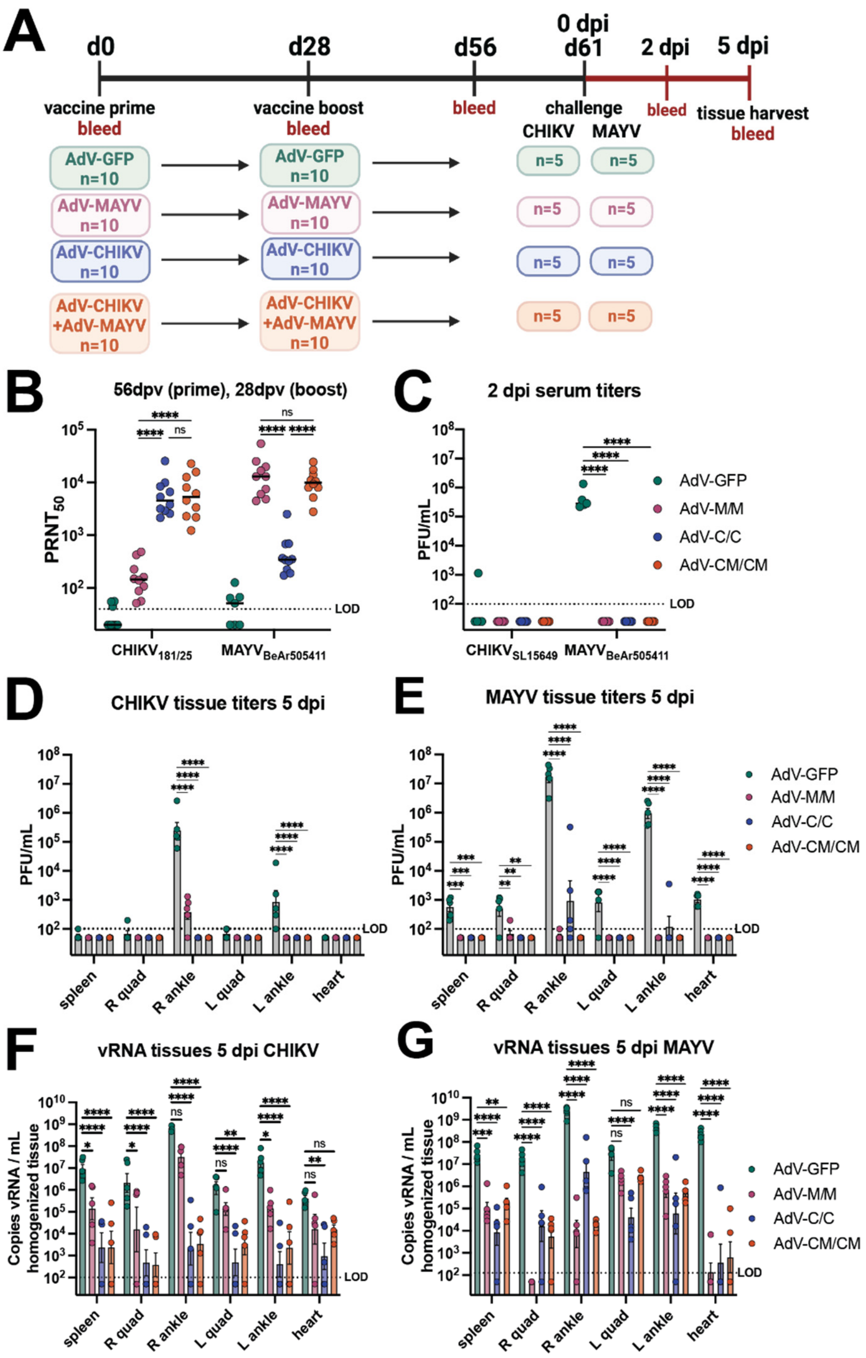

Supplemental Figure S2. Vaccine cross-protection at 5 dpi. (**A**) Study schematic. Ten C57BL/6 mice per adenovirus-vectored (AdV) vaccine group were immunized with intramuscular injections in the left posterior thigh muscle of 10^8 PFU of AdV-CHIKV, AdV-MAYV, or both. At 28 days post-prime, animals received a homologous boost via the same route and dose. Mice were challenged subcutaneously in the right footpad at 61 days post-prime (33 days post-boost) with 10^3 PFU of CHIKV$_{SL15649}$ or 10^4 PFU of MAYV$_{BeAr505411}$. Animals were bled for quantification of serum viremia at 2 days post-infection (dpi) and at 5 dpi spleen, quadriceps, ankles, and heart were harvested for quantification of virus. (**B**) Neutralization data by 50% plaque reduction test (PRNT$_{50}$) against CHIKV$_{181/25}$ and MAYV$_{BeAr505411}$ using sera collected at 56 days post-prime (28 days post-boost). The limit of detection (LOD) for neutralization assays was 40. (**C**) Serum infectious virus detection by limiting-dilution plaque assay on Vero cells measured in PFU/mL. (**D**) Infectious virus isolation in tissue homogenates by plaque assay at 5 dpi for CHIKV and (**E**) MAYV. In (**C**–**E**), the LOD is 100 PFU/mL of tissue homogenate. (**F**) Quantification of viral RNA (vRNA) by qRT-PCR in tissues at 5 dpi for CHIKV and (**G**) MAYV. The LOD for vRNA detection in (**F**,**G**) was 100 copies/μL of tissue homogenate. All statistical analyses are the result of log-transformed, two-way ANOVAs with Dunnett's multiple comparisons where ns $P > 0.05$, * $P \leq 0.05$, ** $P \leq 0.01$, *** $P \leq 0.001$, **** $P \leq 0.0001$. For titer data in (**C**–**E**), only significant comparisons $P < 0.05$ are shown. For neutralization titers, comparisons are not shown to the AdV-GFP group for clarity but every comparison was significant $P \leq 0.05$. For (**B, C**) the median is shown and in (**D**,**E**), bars are mean with SEM.

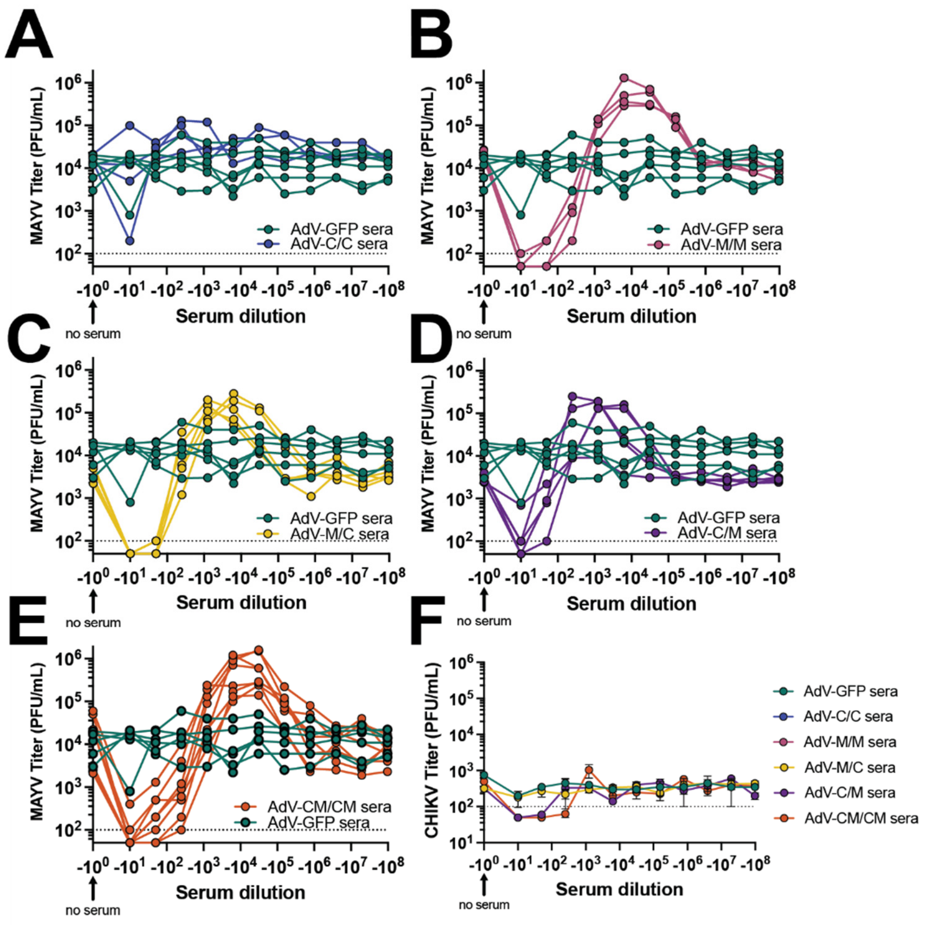

Supplemental Figure S3. Raw titer data for MAYV and CHIKV ADE assays. Related to **Figure 5**. All graphs (**A–F**) are in log-scale reporting raw viral titers (PFU/mL) of RAW264.7 cell supernatants titered in limiting-dilution plaque assays on Vero cells.

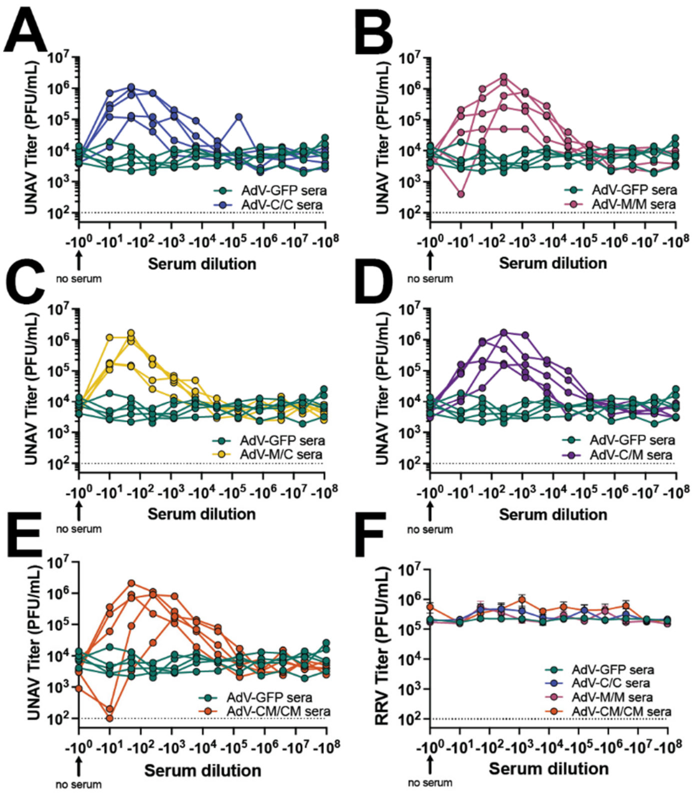

Supplemental Figure S4. Raw titer data for UNAV and RRV ADE assays. Related to **Figure 6**. All graphs (**A**–**F**) are in log-scale reporting raw viral titers (PFU/mL) of RAW264.7 cell supernatants titered in limiting-dilution plaque assays on Vero cells.
